# Does Practicing CSR Makes Consumers Like Your Shop More? Consumer-Retailer Love Mediates CSR and Behavioral Intentions

**DOI:** 10.3390/ijerph14121558

**Published:** 2017-12-12

**Authors:** Ching-Wei Ho

**Affiliations:** Department of Marketing, Feng Chia University, Taichung 40724, Taiwan; cweiho@fcu.edu.tw

**Keywords:** CSR, environmental concern, consumer-retailer love, repeat patronage, willingness to pay

## Abstract

This research paper was designed to examine the influence of corporate social responsibility (CSR) associations and environmental concerns on consumer-retailer love and attitude toward the retailer, as well as the subsequent effects on consumer behavioral intentions regarding the retailer, such as repeat patronage intention and willingness to pay a premium price for products offered by the retailer. In this study, a questionnaire survey was conducted on consumers for the purpose of investigating five proposed hypotheses. This research applied partial least squares (PLS) to exam the hypotheses and analyze the data. The findings of this research indicated that CSR association and environmental concern both have positive effects on consumer-retailer love and attitude toward the retailer. Also, the results showed that consumer-retailer love has a significantly positive effect on consumer attitude towards the retailer. This paper establishes that consumer-retailer love and attitude toward a retailer are main mediators of the relationship between CSR associations, environmental concern, and consumer behavioral intentions.

## 1. Introduction

Corporate social responsibility (CSR) is a critical thought in the scholastic domain, and is becoming an urgent issue on actual corporate agendas [1,2,3]. Concepts of CSR ranges are variable; for example, Perrini et al.’s [4] research divided CSR into consumer-oriented and environmentally-oriented concepts. For the consumer-oriented concept, a great deal of researchers proposed that firms can obtain huge benefits when they are seen as being socially responsible by their stakeholders [5,6], particularly their consumers [7,8], namely as a company with CSR associations [9]. Meanwhile, the environmentally-oriented concept refers to respecting and protecting the natural environment [4], essentially a company with environmental concern. Several studies have addressed that consumers’ environmental concern influence purchase behavior of environmentally sound products [10,11]. Hansla et al. [12] also provided demonstration of environmental concern’s direct and indirect influences on consumers’ willingness to purchase green products at a premium price. 

Previous academic research (e.g., [13,14,15,16]), has shown that CSR has a positive effect on consumers’ assessments and companies’ purchase intentions. The positive relationship between CSR and consumer patronage makes company administrators recognize that CSR is not only an ethical/ideological imperative, but also an economic one in today’s marketplace [17]. One of the fundamental ideas about its influence is that CSR allows consumers to have positive attitudes towards the company and its offerings, including beliefs about its honesty, consumer responsiveness, and truth in advertising [18]. Furthermore, CSR also permits consumers to associate with a specific corporate brand to define themselves. Therefore, when consumers identify that the CSR belief has certain features that overlap with their own self-concepts, they evolve a superior brand attachment and love for the company and are more likely to support it [19]. Additionally, in agreement with Perrini et al.’s [4] research, the success of a retailer in marketing its retail brand with CSR associations and environmental concern could be measured by behavioral intentions, for example, repeat patronage intention and willingness to pay. Therefore, the current study is going to explore the consumer evaluation of a retailer by these two kinds of consumer behavioral intentions. 

This empirical study will enhance the knowledge and the relationship among CSR associations, environmental concern, and consumer behavioral intentions in retailing, then examine the mediating effects of attitude toward the retail store and consumer-retailer love. This study hypothesizes that the greater the commitment of a retailer to CSR associations and environmental concern, the greater the level of positive attitude toward the retailer, or consumer-retailer love, and the greater level of re-patronage intention or willingness to purchase. This study initiates by building a framework to picture how CSR associations and environmental concerns affect attitude toward the retailer and consumer-retailer love, and how consumer behavioral intentions are influenced by the mediating effects of attitude and consumer-retailer (C-R) love. Additionally, this study investigates the model and hypotheses using structural equation modeling with survey data. Lastly, this research concludes with an argument about marketing connotations, as well as theoretical and practical implications, and limitations for further investigation.

## 2. Literature and Hypothesis

### 2.1. CSR Associations and Environmental Concern

Corporate social responsibility (CSR) associations have been identified as one of the basic categories of corporate associations [9]. According to del Bosque [20], the concept of corporate association evolved during psychology investigations, and is referred to as all the information that a person gathers about a company. Therefore, associations of corporate social responsibility refer to the perceived image consumers have about the social character of the company [9]. 

Actually, more and more studies have tried to express what it means for a firm to be socially responsible, as CSR is an essential construct in the scholastic domain, and has become an urgent issue for actual corporate agendas [1,2,3]. Among the numerous definitions of corporate social responsibility that have been adopted, the one most often cited is from Carroll [21], which structured a four-part conceptual model, comprised of economic, legal, ethical, and philanthropic responsibilities. In accordance with Tian et al. [8], economic and legal responsibilities are the fundamental level of CSR that must be achieved by corporations without question, so the existing research mainly discusses CSR practices at higher levels, namely, the corporations’ ethical and philanthropic responsibilities. Ethical responsibilities include those standards, norms, or expectations that reveal a concern for stakeholders, for example, the environment, civil rights, and many other forms, while philanthropic responsibilities embody those corporate actions that respond to society’s expectations, such as business contributions to the arts, education, or the community [22].

For a long time, a main subject for business has been whether or not a corporation should be concerned with issues other than profitability [23]. Many scholars suggest that firms can derive huge benefits when they are recognized as being socially responsible by their stakeholders [5,6]. Carroll [22] argued that a socially responsible corporation should attempt to make a profit, obey the law, be ethical, and be a good corporate citizen. These studies highlight that a socially responsible corporation should have concerns beyond just short-term profitability. Furthermore, the concept of CSR also has been defined to suggest that companies integrate social and environmental concerns into their business operations, and conduct their interactions with their stakeholders on a voluntary basis [24]. A business that is environmentally responsible will try to avoid pollution, decrease environmental damage that their products may cause, and in general be sustainable in its operations [25,26]. Accordingly, in this study, CSR associations reveal a company’s status and activities with respect to its perceived social obligations [9,19], such as concern for consumers, employees and corporate philanthropy [20], and environmental concern reflects the commitment by a company to respect and protect the natural environment [4]. 

Numerous marketing research studies have described that CSR behaviors can positively influence consumer attitudes towards a company and its offerings [7,14,23,27,28,29]. Additionally, Murray and Vogel [18] mentioned that CSR activities result in improved attitudes towards the firm, comprising beliefs about its honesty, consumer responsiveness, and truth in advertising, among other issues. Regarding Brown and Dacin’s [9] argument, CSR does not provide information about the attributes or overall quality of the product offered by a firm/store, but it could form an evaluative context for consumers’ more specific firm/store evaluations. Considering the term “generalized customer” suggested by Daub and Ergenzinger [30], customers who are not only consumers, but also actual or potential members of other stakeholder groups, are likely to feel more satisfied and pass a positive evaluative judgment on products or services offered by socially responsible companies/stores. Therefore, in line with previous researches, we propose that a retail store with CSR associations and environmental concern positively influences consumer attitudes toward the retailer. The following two hypotheses are proposed:

**Hypotheses** **1a** **(H1a).** CSR associations positively influence attitude toward the retailer.

**Hypotheses** **1b** **(H1b).** Environmental concern positively influences attitudes toward the retailer.

### 2.2. Consumer-Retailer Love

Most previous researchers and retail managers used the relationship marketing paradigm in order to develop consumer relationships with firms and retailers, especially in the satisfaction-loyalty chain [19]. However, loyalty to services remains elusive and unpredictable [31]. Therefore, apart from the well-accepted satisfaction-loyalty paradigm, another method for studying consumer relationships with retailers focuses on feelings of attachment and love, (e.g., [32,33]). Meanwhile, the term “brand love” has been used in marketing literature by previous scholars (e.g., [32,34,35,36]), and has been described as the phenomenon of consumers forming close, affection-laden relationships with brands [19]. 

The research on brand love is relevantly new, but seems to attract increasingly growing interest in both academia and practice [37]. One of the first studies about brand love was by Shimp and Madden [38], who established a typology of consumer-object relationships based analogously on the triangular theory of love [39]. Recently, Fournier [40] suggested the significance of love in consumers’ relationships with brands. Moreover, Kleine and Baker [41] discussed the bonds that people form with material possessions, positing material possession attachment as a significant mechanism used by consumers to valuate goods. Likewise, Ahuvia [34], applying an interpretive paradigm, tested the role of loved objects and activities on our sense of who we are. Carroll and Ahuvia’s [32] and Ahuvia’s [34] works were among the first to empirically identify the concept and antecedents of consumer-firm love. Recently, Kim et al. [42] examined customer love in the context of apparel and grocery retailing, and the empirical results showed that consumer-retailer love was ultimately leading to action loyalty. Therefore, the study here applies Carroll and Ahuvia’s [32] concept of consumer-firm love in a service setting, involving grocery retailers, to define consumer-retailer love as the degree of passionate emotional attachment that a satisfied consumer has for a particular retailer. 

It should be noted that consumer ties of affection with service businesses still hardly receive any research attention [43]. Vlachos and Vrechopoulos [19] indicate that corporate social responsibility (CSR) is a direct antecedent of consumer-retailer love. CSR is about doing social and environmental good; consumers may use associations with corporate responsibility to define themselves and retain a positive inner and social self-image. CSR is based not only on ethics, but also on enlightened self-interest [44,45]. Patronizing a socially responsible retailer might make consumers feel better about themselves, helping customers understand who they are as people, and ultimately satisfying self-definitional and self-expressive needs [19]. Therefore, it is likely that CSR (including CSR associations and environmental concern) positively influences consumer-etailer love levels. Therefore, the following hypotheses are proposed:

**Hypotheses** **2a** **(H2a).** CSR associations positively influence consumer-retailer love.

**Hypotheses** **2b** **(H2b).** Environmental concern positively influences consumer-retailer love.

Additionally, consumers may feel that the retailer is doing social and environmental good on their behalf. Having psychologically defined themselves as a member of the retailer, consumers then internalize the retailer’s stereotypical norms as personal norms, which reveals positive attitudes toward the retailer [46,47,48]. Accordingly, the following hypothesis is suggested:

**Hypotheses** **3** **(H3).** Consumer-retailer love positively influences attitude toward the retailer.

### 2.3. Behavioral Intentions

According to Perrini et al. [4], the success of a retailer in marketing its retail brand with CSR associations and environmental concern could be measured by two kinds of consumer behavioral intention: repeat patronage intention and willingness to pay a premium price. Repeat patronage intention refers to customer intention to further patronize the service provider/retail store, as well as the intention to increase both the scale and scope of the customer’s relationship with the retailer [49]. In this study, consumer repeat patronage intention is at the retail store level, which translates into preference for a retail store with CSR associations and environmental concern over other retailers. 

Some previous research, (e.g., [49]), has demonstrated that attitudinal loyalty is positively related to behavioral intentions. Social exchange theory supports a link between customer attitudinal evaluations and customer behavior intention. Social exchange theory explains one party’s feelings about a relationship with another party as depending on the first party’s perceptions of fairness in a process of negotiated exchanges [50,51]. When a business engages in CSR associations and environmental concern, consumers may perceive the company to be altruistic, which could lead to more favorable evaluations of the company [52]. Therefore, consumers are more likely to engage in reciprocation that may benefit the company [53]. Hence, the research proposes the hypothesis as follows:

**Hypotheses** **4** **(H4).** The more positive the attitude toward the retailer, the more likely that a consumer will exhibit (a) the repeat patronage intention and (b) willingness to pay a premium price.

Meanwhile, willingness to pay (WTP) is defined as the maximum price a customer is likely to pay for a product or service under the given circumstances of time and place [54,55]. In this study, consumer willingness to pay a premium price is for the retail product level, because the CSR-oriented product, e.g., the organic product, is retailed at a higher premium than comparative non-CSR goods. Some works (e.g., [32,43,56]), have posited that consumer-retailer love has a direct impact on consumer re-patronage intentions. Also, the impact of consumer-retailer love on consumer willingness to purchase is well documented [19]. Hence, the following hypothesis is suggested:

**Hypotheses** **5** **(H5).** Consumer-retailer love positively influences (a) the repeat patronage intention and (b) willingness to pay a premium price.

**Figure 1:**
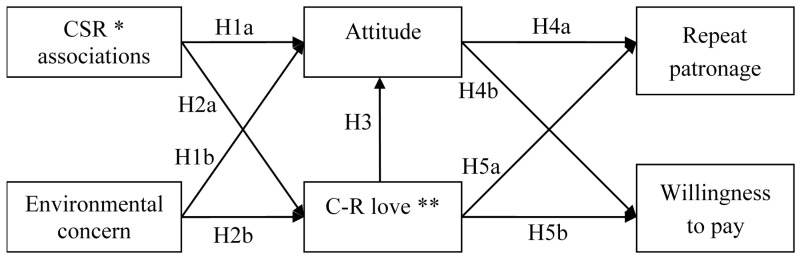
The theoretical model. *: corporate social responsibility. **: consumer-retailer love.

The theoretical model that incorporates all these hypothesized relationships is illustrated below in Figure 1. 

## 3. Research Methodology 

### 3.1. Data Collection and Sampling

To test our hypotheses empirically, we choose an organic retailer in Taiwan as the study case, because the concept of “organic” is labeled and perceived as concerning consumers and protecting the environment by Taiwanese people. The development of the Taiwanese organic market is just over a decade old. The market for organic products is still undeveloped and just a niche, but consumer interest is growing. Taiwanese consumer demand for organic goods is concentrated in the main metropolitan areas, and organic goods are retailed mostly by chain specialty retailers or directly by farmers. Therefore, the data used to examine these hypotheses were collected from organic product purchasers, using a structured questionnaire developed for this research and adapting others used in earlier studies. The questionnaire was randomly distributed outside stores in the city center of Taichung, where almost all main chain organic retail brands, such as Leezen, Santa Cruz, and Earthlife, are located. All these three firms are commercial chains established over the past 20 years. Beyond selling organic products, their CSR practices include holding a series of events/campaigns for protecting trees, establishing an organic agricultural foundation for promoting natural farming, cooperating with the Aboriginal and small farmers to help them create better quality products, and other environmental protection issues. All of their information and activities are published on their websites and can be found by the public easily.

Every fifth consumer was asked to participate in the study, after a random starting point. We asked participants to keep in mind the organic shop and the products they have experienced or consumed while answering the questions. Participants were guaranteed confidentiality and anonymity. Self-administered questionnaires, with assistance from the researchers as needed, were used to ensure a better response rate and reduce non-sampling bias throughout the survey process. An effort was made to randomize the data collection at different times of the day and week. According to Chin and Newsted [57], a sample size of 150–200 is required to attain reliable coefficient values using partial least squares (PLS) analysis [58]. It was suggested that the ratio of observations to the independent variable should not fall below five (5:1) [59], although the preferred ratio is 10 respondents for each independent variable (the minimum ratio of observation to variables is 10:1) [60]. Therefore, bearing in mind the 18 variables that need to be used in structural equation modeling (SEM), this research required a minimum sample size of 180 respondents. At the end of the data collection period, 208 questionnaires had been distributed, of which 197 effective questionnaires were returned for data analysis.

### 3.2. Measurement of the Variables

The survey questionnaire was established by adapting the measurements from a variety of studies. CSR associations were measured using three items adapted from Perrini, et al. [4] and Tian, et al. [8]. Environmental concern was measured using three items adapted from Perrini, et al. [4]. To measure consumer-retailer love, we employed the three-item scale suggested by Vlachos and Vrechopoulos [19], and to measure attitude toward the retailer, we applied the three-item scale suggested by Mandhachitara and Poolthong [49]. The measurement of the repeat patronage intention construct was comprised of three items, also adapted from the works of Mandhachitara and Poolthong [49]. The measurement for the willingness to pay a premium price construct consisted of three items adapted from Chaudhuri and Holbrook [61] and Perrini et al. [4]. All items that were employed to measure the constructs used a five-point Likert scale, demonstrating the extent of agreement or disagreement with the item. The items for each construct and their measurement scales are shown in Table 1. 

### 3.3. Data Analysis

The research applied partial least squares (PLS) to exam its hypotheses and to analyze the data. The PLS algorithm permits each indicator to vary in terms of how much it contributes to the composite score of the latent variable, instead of assuming equal weight for all indicators of a scale (Chin et al. 2003 [62]; Hur et al. 2011 [58]). As stated by Anderson and Swaminathan [63], PLS path modeling is normally applied in information systems studies [64,65], marketing [66], and international business [67], which necessitates simultaneous estimation of the factor loadings of the measurement model and path coefficients of the structural model. The research applied PLS rather than other SEM approaches (i.e., Amos or LISREL), since the PLS approach puts minimal restrictions on sample size and residual distribution [58,68].

## 4. Findings

### 4.1. Demographic Profile of Respondents

Of the 197 respondents surveyed, a total of 53% were female, and 47% were male. Regarding age, 25% were 30–39 years old, and 24% were 40–49 years old. These two groups thus accounted for the biggest percentage of the sample, followed by those aged 50–59 years (22%), 20–29 years (21%), 60 years and above (2%), and under 20 years (1%). The majority of the respondents had a university degree (59%) and a graduate degree (38%). In terms of monthly income (NT$), 44% had 30–50 K, 27% had less than 30 K, 17% had 50–80 K, and 12% had more than 80 K. 

### 4.2. Measurement Model

The two-step method was applied in the research, as described by Anderson and Gerbing [69]. First, we evaluated reliability and convergent validity as presented in Table 1, and then discriminant validity, as shown in Table 2. When examining reliability, Cronbach’s alpha showed that all constructs presented a value above 0.6 (adopted by Bagozzi and Yi [70]). To test for convergent validity, composite reliability (CR), factor loading, and average variance extracted (AVE) were checked. These measures were considered acceptable if an individual item loading was more than 0.7, CR exceeded 0.7, and AVE was more than 0.5 [71]. 

In order to inspect the discriminant validity of the constructs, the research addressed the Fornell and Lacker [72] criterion, where the average variance shared between each construct and its measures should be bigger than the variance shared between one construct and other constructs. As revealed in Table 2, the correlations for each construct were less than the square root of AVE for the indicators’ measuring that construct, thus indicating adequate discriminant validity. 

### 4.3. Structural Model

**Figure 2:**
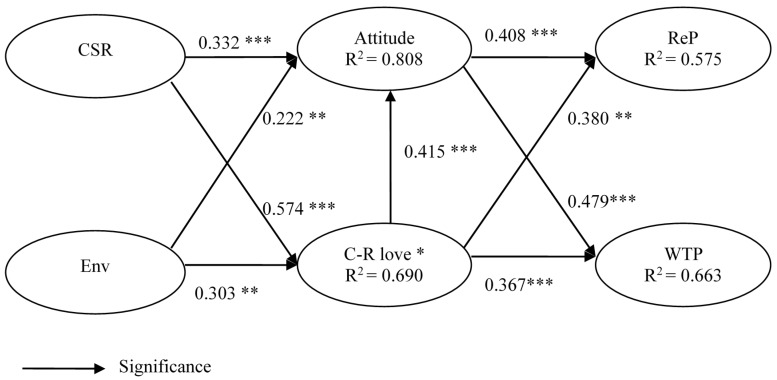
Results of the structural model analysis. Note: *: consumer-retailer love; **: *p* < 0.01; ***: *p* < 0.001.

By looking at the R^2^ values, the explanatory power of the structural model was evaluated. The R^2^ values ranged from 0.575 to 0.808 (in Figure 2), which proposes that the modeled variables can explain 57.5% to 80.8% of the variance of the respective dependent variables.

In Figure 2, CSR associations exerted a significant and positive influence on both attitudes toward the retailer (H1a, β = 0.332, *p* < 0.001) and C-R love (H2a, β = 0.574, *p* < 0.001). Meanwhile, environmental concern had a significant and positive influence on both attitude toward the retailer (H1b, β = 0.222, *p* < 0.05) and C-R love (H2b, β = 0.303, *p* < 0.005). Hence, H1 and H2 were fully supported. Meanwhile, consumer-retailer love had a positively significant influence on attitude toward the retailer (H3, β = 0.415, *p* < 0.001). Further, attitude toward the retailer had a significant and positive influence on both re-patronage intention (H4a, β = 0.408, *p* < 0.001) and willingness to pay a premium price (H4b, β = 0.479, *p* < 0.001). Meantime, consumer-retailer love had a significant and positive influence on both re-patronage intention (H5a, β = 0.380, *p* < 0.05) and willingness to pay a premium price (H5b, β = 0.367, *p* < 0.001). Therefore, both H4 and H5 were fully supported. 

The nine paths that were surveyed in the structural model are summarized in Table 3 below.

## 5. Discussion

The model proposed in the current study examined the effect of CSR associations and environmental concern on consumer-retailer love and the attitude toward the retailer, as well as the subsequent effects on consumer behavioral intentions towards the retailer, such as repeat patronage intention and willingness to pay a premium price on products offered by the retailer. The findings achieved from the study provide significant contributions to and have essential implications for both marketing academia and practitioners. 

In previous studies, the issues of CSR were always discussed while either examining CSR as a whole (e.g., [8,20,49]), or dividing it into consumer and environmental CSR (e.g., [4]). However, the concept of CSR includes many different components, and it is not detailed enough to examine CSR as a whole, nor is it enough to examine it just from the consumer and environmental sides. Therefore, this current study divided CSR into CSR associations and environmental concern. CSR associations include the CSR activities of a retailer regarding the concern of consumers, society, and corporate philanthropy, and the environmental concerns reflect the CSR activities of a retailer regarding the protection of the natural environment. The findings reveal that CSR association and environmental concern both have positive effects on consumer-retailer love and attitudes toward the retailer. That is, the more a retailer is perceived as caring about consumers, society, and the natural environment, the more likely it is that consumers will have positive attitudinal responses and passionate emotional attachment for that particular retailer. These results are in some ways consistent with previous researches [19,49,73]. 

Additionally, when talking about the consumer behavioral intentions in retailing, previous literature usually discussed the intention toward the retailer’s products, i.e., the purchase intention (e.g., [4,8,19,73]), or the intention to the retail store, i.e., re-patronage intention (e.g., [49]). It is not easy to find a study that explores those two behavioral intentions in the same research. Actually, it is very important to recognize that consumer loyalty or intentions are toward the level of products offered by the retailer, or the level of retail store. Therefore, this empirical study investigated both behavioral intentions and found that positive perception of CSR associations and environment efforts not only left consumers with a positive attitude and emotional attachment toward the retailer, but also generated positive behavioral intention at both product and store levels. 

This finding also indicates that C-R love has a significantly positive effect on consumer attitude towards the retailer. That is to say, when consumers recognize that the CSR belief or concept has certain traits that overlap with their own self-concepts, they will develop a more positive attitude towards the particular retailer, and will be more likely to support it. This direction of causality has not been found before in the literature, and thus can be seen as pioneering, setting a new and important benchmark for further study. 

The role of C-R love and attitude towards the retailer as an antecedent of both consumer behavioral intentions (re-patronage intention to a retail store and willingness to pay a premium price for a retailer’s product) in the field of CSR has been addressed very few times. This finding pointed out that a higher degree of C-R love and a more positive attitude toward the retailer seem to be linked to stronger consumer intentional responses, to both retailers’ products and stores. 

Moreover, this study establishes that C-R love and attitude toward the retailer are both key mediators of the relationship between CSR associations/environmental concerns, and consumer behavioral intentions. From Figure 2, in the relationship between CSR associations and consumer behavioral intentions, the mediation effect of C-R love is stronger than that of the attitude towards the retailer (β = 0.218 > β = 0.135; β = 0.211 > β = 0.159). Also, there is a similar situation in the relationship between environmental concern and consumer behavioral intentions, where the mediation effect of C-R love is stronger than that of the attitude towards the retailer (β = 0.115 > β = 0.091; β = 0.111 > β = 0.106). 

With regard to consumer behavioral intentions, this research discloses that the strongest effect for repeat patronage intention to a retail store is the path of CSR associations through C-R love (β = 0.218), whilst the weakest effect is the path of environmental concern through attitude towards the retailer (β = 0.091). Furthermore, the strongest effect on willingness to pay a premium price for a retailer’s product is through the path of CSR associations through C-R love (β = 0.211), whereas the weakest effect is through the path of environmental concern through attitude towards the retailer (β = 0.106).

Finally, an interaction was found between consumers and retailers of organic products. Consumers of organic products are more aware of the need to support CSR. Retailers of organic products are more prone to implement CSR practices. The fact that the majority of respondents had a university degree (59%) and above (38%) suggests that the clientele of this sample has a high cultural level, and thus, is more informed than the average clientele on the relevance of organic products and CSR.

## 6. Conclusions

### 6.1. Managerial Implications

The results from this research have practical implications for commercial practices. Firstly, it is clear that both CSR associations and environmental concern have the potential to exert a significant positive impact on consumer evaluation. However, from a managerial point of view, these results should be considered supportive of those retailers that have chosen to engage in CSR activities and environmental concern. In reality, CSR has been viewed as a means to improve public relations, develop corporate image, and increase sales. Although both CSR associations and environmental concern have significant positive influences on consumer attitudes and C-R love, the effect of CSR associations is still stronger than that of environmental concern. This study shows that Taiwanese consumers care more about CSR activities, with regard to caring about consumers and society, than the natural environment. Therefore, retail managers could make every effort to address consumers’ needs and rights, and aim at contributing to society, in order to obtain greater C-R love and positive attitude towards their corporation. 

The results also provide new insights for practitioners, in order to enable them to create and maintain a state of closeness between a CSR retailer and consumer response (behavioral intentions), through developing consumer-retailer love, which is more effective than forming consumers’ attitudes toward the retailer by themselves. Namely, consumers might use associations with a retailer’s CSR to label themselves, and CSR is based on enlightened self-interest ([44,45]). Therefore, marketing managers of CSR retailers should pay closer attention to consumers’ needs and wants, and focus on consumers’ feelings of attachment and love towards the retailer. 

Finally, this study provides another new customer insight for retail store managers and retail merchandise managers. Store managers always care about whether consumers re-patronize their store, and merchandise managers care about whether a consumer is willing to pay more for a product with CSR concerns (e.g., organic products) offered by a CSR retailer. These findings show that when consumers evaluate whether to patronize a retailer again, and pay a premium price for a CSR retailer, the path of CSR associations via C-R love play a more important role than other paths. Therefore, at the store level and the product level, managers should focus on developing their retailer’s CSR activities, with regard to the protection of the consumers’ society, and help customers who shop at their store recognize who they are as people, to satisfy self-definitional and self-expressive needs, ultimately achieving re-patronage of the store and willingness to pay for premium priced products. 

### 6.2. Limitations and Future Research

Certain limitations of this research should be noted. Alongside these limitations, we will also recommend directions for further study. Firstly, the research was carried out in Taiwan, thereby restricting its findings to that culture. Recent study has proposed that cognitive, emotional, and behavioral responses may vary across cultures [74]. Based on Hofstede’s [75] cultural dimensions, the Taiwanese culture is known to be more collective than individualistic. Therefore, the influence of behavioral intentions might be different in more individualistic cultures. Further studies would discover the role of perception and behavior in terms of consumer responses to CSR across a variety of cultures.

Secondly, this research adopted attitudes toward retailers and consumer-retailer love as antecedents of behavioral intentions. Other relational variables, such as satisfaction or trust, should also be considered and tested in the relationships among antecedents, and their effects on consumer intentions. 

Thirdly, this study divided consumers’ responses to retailers into two kinds: re-patronage at the store level and willingness to pay at the product level. According to Ho and Temperley’s [76] summary, retail includes three levels: the retail corporate (brand of corporate), the retail store (brand of store), and the retail product (brand of item). Therefore, further research could try to adopt Dawson’s three-level retail model. Moreover, other behavioral variables, such as in/ex-role behaviors, could also be considered and examined in the future. 

Additionally, this finding indicates that C-R love has a significantly positive effect on consumer attitude towards the retailer. This direction of causality could be seen as pioneering, setting a new and important benchmark for future study. Therefore, it could also be argued that the direction of causality is actually the reverse, i.e., that consumer attitude toward the retailer causes C-R love. 

In this study, the sample consists of organic grocery retailers. The clientele of organic products could be willing to pay higher prices due to two reasons: the products themselves, and the CSR practices of these firms. Therefore, at this point, the use of organic grocery retailers may affect the “willingness to pay,” because CSR and the healthier properties of organic products are interwoven with one another. At the current stage, it is discussed, in general, as a whole, but these two items should be separated in future research.

Finally, this study examined only organic grocery retailers. Future research should examine the modeled relationships and influences in different retail sectors, with different types of CSR activities.

## Figures and Tables

**Table 1:** The constructs and their measurement items.

Construct	Measurement Items	Loading	α *	CR **	AVE ***
CSR associations (CSR)	X’s CSR actions sincerely aimed at contributing to society.	0.863	0.87	0.92	0.80
	X took a lot of effort to be socially responsible.	0.909			
	X protects consumers’ rights.	0.908			
Environmental concern (Env)	X cares for the natural environment.	0.921	0.89	0.93	0.82
	X is well established about environmental concern.	0.899			
	X is sensitive to ecological issues.	0.904			
Consumer-retailer love (C-R)	X is “my” store; my favorite retailer that I can count on.	0.947	0.94	0.96	0.89
	If I were describing myself, shopping at X would likely be something I would mention.	0.952			
	I always enjoy shopping at X.	0.936			
Attitude (Att)	I will say positive things about X.	0.938	0.93	0.95	0.88
	I always consider X as my first choice.	0.940			
	I give a high valuation to X.	0.930			
Repeat patronage (ReP)	I will definitely keep shopping at X.	0.907	0.92	0.95	0.86
	If there is another retailer’s environmental performance as good as X, I prefer to shop at this retailer next time.	0.945			
	I shall continue considering X as my main store in the next few years.	0.932			
Willingness to Pay (WTP)	Buying X’s product seems smart to me even if they cost more.	0.947	0.94	0.96	0.89
	I’m ready to pay a higher price for X’s products.	0.955			
	I’d still buy X’s products if other products reduced their prices.	0.929			

*: Cronbach’s alpha. **: composite reliability. ***: average variance extracted.

**Table 2:** Correlation matrix.

	Mean	SD	CSR	Env	CR	Att	ReP	WTP
CSR	3.905	0.860	0.89					
Env	3.900	0.924	0.78	0.91				
CR	3.829	0.849	0.81	0.75	0.94			
Att	4.020	0.837	0.84	0.79	0.85	0.94		
ReP	3.658	0.966	0.65	0.68	0.73	0.73	0.93	
WTP	3.766	0.963	0.73	0.73	0.77	0.79	0.73	0.94

Note: Diagonals represent the square root of the average variance extracted, while the other entries represent the correlations.

**Table 3:** Results of testing.

Hypothesized Relationship		Coefficient	*t*-Value	Supported
H1a	CSR→Attitude	0.332 ***	3.252	Yes
H1b	Env→Attitude	0.222 **	2.656	Yes
H2a	CSR→C-R love	0.574 ***	5.465	Yes
H2b	Env→C-R love	0.303 **	2.762	Yes
H3	C-R love→Attitude	0.415 ***	3.983	Yes
H4a	Attitude→ReP	0.408 ***	3.259	Yes
H4b	Attitude→WTP	0.479 ***	4.316	Yes
H5a	C-R love→ReP	0.380 **	2.844	Yes
H5b	C-R love→WTP	0.367 ***	3.159	Yes

Note: ** *p* < 0.05; *** *p* < 0.001.
